# Supplementary material for: Spatio-temporal quantile regression analysis revealing more nuanced patterns of climate change: A study of long-term daily temperature in Australia
Source: PLoS One. 2022 Aug 24;17(8):e0271457. doi: 10.1371/journal.pone.0271457 (PMC9401128; doi:10.1371/journal.pone.0271457)
Supplement: S1 File — This supplementary document includes four sections, i.e., S1. Additional Figures for Exploratory Analysis, S2. Results of Inter-annual Variance Model, S3. Simulation Study and S4. Result of Quantile Trend by Season. (PDF) [file pone.0271457.s001.pdf]

Supplementary Document

S1.Additional Figures for Exploratory Analysis

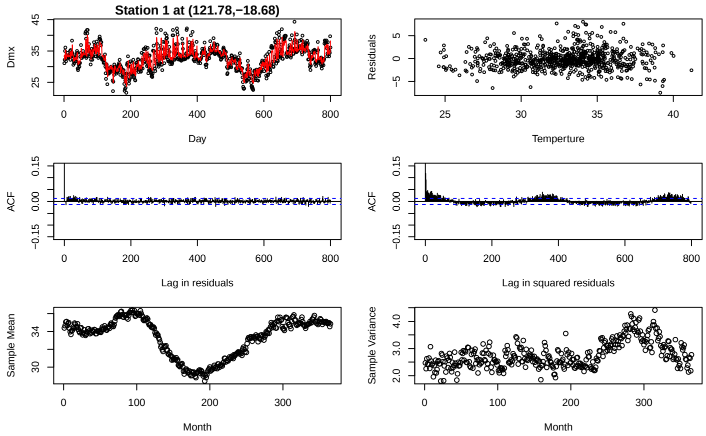

Figure 1: Exploratory analysis for station 1. Top left: fitting parametric mean model for Dmx. Top right: residuals of fit against predicted Dmx for the parametric mean model. Middle left: Auto-Correlation Function (ACF) plot for residuals for lag up to 800 days. Middle right: ACF plot for squared residuals for lag up to 800 days. Bottom left: sample mean over 60 years. Bottom right: sample variance over 60 years.

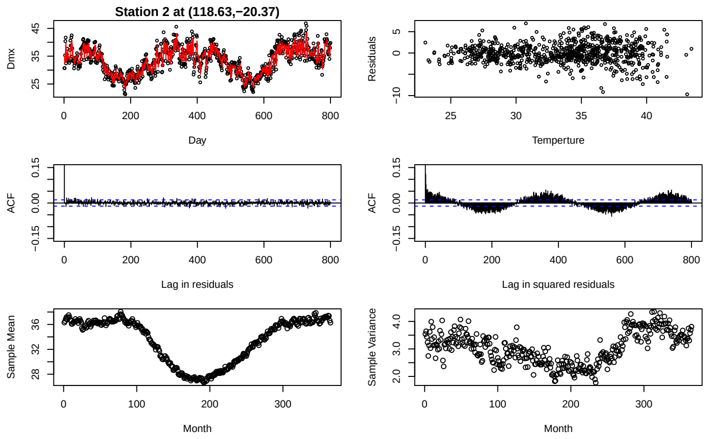

Figure 2: Exploratory analysis for station 2. Top left: fitting parametric mean model for Dmx. Top right: residuals of fit against predicted Dmx for the parametric mean model. Middle left: Auto-Correlation Function (ACF) plot for residuals for lag up to 800 days. Middle right: ACF plot for squared residuals for lag up to 800 days. Bottom left: sample mean over 60 years. Bottom right: sample variance over 60 years.

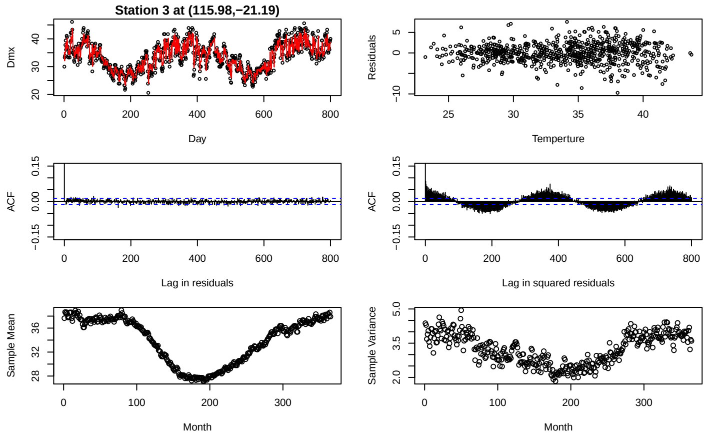

Figure 3: Exploratory analysis for station 3. Top left: fitting parametric mean model for Dmx. Top right: residuals of fit against predicted Dmx for the parametric mean model. Middle left: Auto-Correlation Function (ACF) plot for residuals for lag up to 800 days. Middle right: ACF plot for squared residuals for lag up to 800 days. Bottom left: sample mean over 60 years. Bottom right: sample variance over 60 years.

S2. Results of Inter-annual Variance Model

We model the variance $\sigma_d^2(\mathbf{s})$ as the following five models. Note that the fourth order truncated Fourier series is suggested in [4, 1, 3, 2, 6].

Model 1

$$\sigma_d^2(\mathbf{s}) = \beta_0(\mathbf{s}) + \beta_1(\mathbf{s})\mu_d(\mathbf{s}) + \beta_2(\mathbf{s})\mu_d^2(\mathbf{s}). \tag{1}$$

Model 2

$$\sigma_d^2(\mathbf{s}) = \beta_0(\mathbf{s}) + FS4(d) \tag{2}$$

Model 3

$$\sigma_d^2(\mathbf{s}) = \beta_0(\mathbf{s}) + \beta_1(\mathbf{s})\mu_d(\mathbf{s}) + \beta_2(\mathbf{s})\mu_d^2(\mathbf{s}) + FS4(d, a(\mathbf{s}), b(\mathbf{s})) \tag{3}$$

Model 4 (AR(1))

$$\sigma_d^2(\mathbf{s}) = \beta_0(\mathbf{s}) + \beta_1(\mathbf{s})\mu_d(\mathbf{s}) + \beta_2(\mathbf{s})\mu_d^2(\mathbf{s}) + FS4(d) + \rho_1(\mathbf{s})(\hat{\sigma}_{d-1}^2(\mathbf{s}) - \sigma_{d-1}^2(\mathbf{s})) \tag{4}$$

Model 5 (AR(2))

$$\sigma_d^2(\mathbf{s}) = \beta_0(\mathbf{s}) + \beta_1(\mathbf{s})\mu_d(\mathbf{s}) + \beta_2(\mathbf{s})\mu_d^2(\mathbf{s}) + FS4(d) + \sum_{k=1}^{2} \rho_k(\mathbf{s})(\hat{\sigma}_{d-k}^2(\mathbf{s}) - \sigma_{d-k}^2(\mathbf{s})) \tag{5}$$

These five models are fitted for all the selected stations and three criteria are used for model selection, i.e., AIC, BIC and AICc, according to [5]. We also take logarithm transformation on $\sigma_d^2(\mathbf{s})$ and compare these above five models.

Table 1 shows the summary of model selection without taking logarithm transformation over inter-annual variance and Table 2 shows the results of taking logarithm transformation. Generally, model 4 and 5 outperform the first three models. However, these models without taking logarithm transformation are not able to account for all heterogeneity in variance. As shown in Figure 4, the residuals of fitted models still have clear pattern, which means there still is unaccounted heterogeneity in variance. While Figure 5 shows that taking logarithm transformation can improve the performance of models.

Table 1: Number of stations that a model performed best under a criteria

Data	Creteria	Model 1	Model 2	Model 3	Model 4	Model 5
Daily maximum	AIC	0	0	0	35	38
	BIC	0	0	3	56	15
	AICc	0	0	0	36	37
Daily minimum	AIC	0	0	1	53	19
	BIC	1	2	3	63	4
	AICc	0	0	1	53	19

Table 2: Number of stations that a model performed best under a creteria (Logarithm transformation)

Data	Creteria	Model 1	Model 2	Model 3	**Model 4**	Model 5
Daily maximum	AIC	0	0	0	45	28
	BIC	0	0	1	68	4
	AICc	0	0	0	45	28
Daily minimum	AIC	0	0	1	59	13
	BIC	5	4	3	57	4
	AICc	0	0	1	60	12

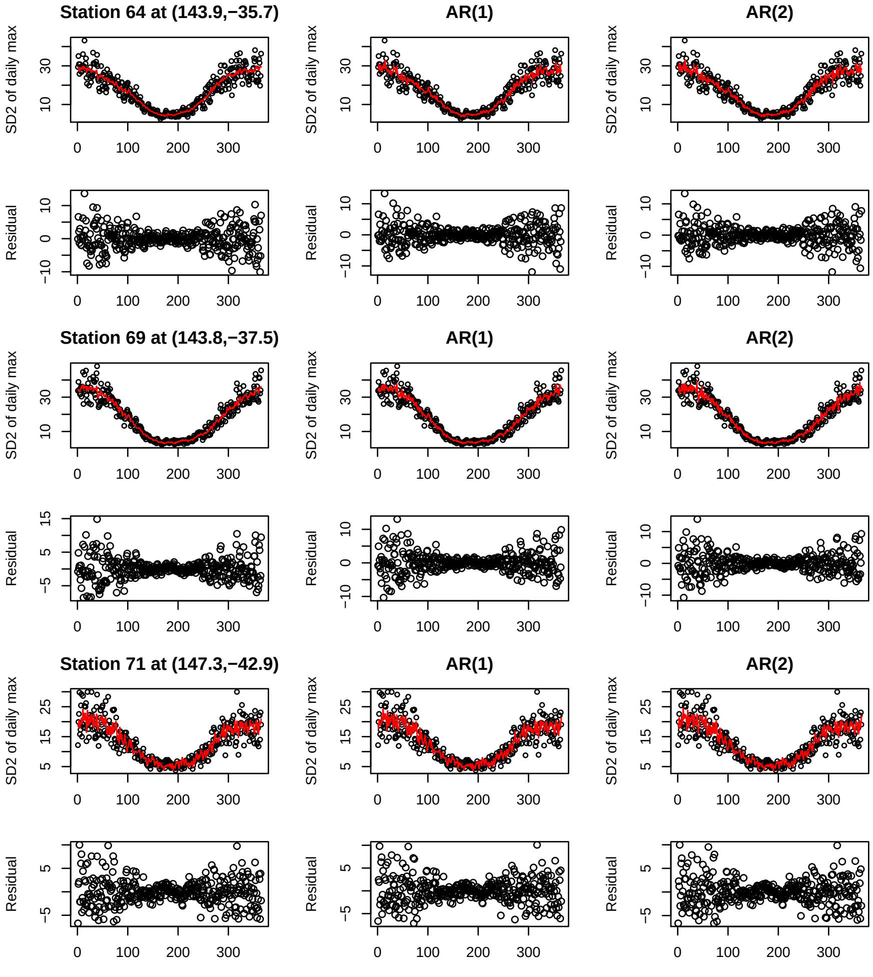

Figure 4: Fitting results of inter-annual variance with model 3, 4 and 5.

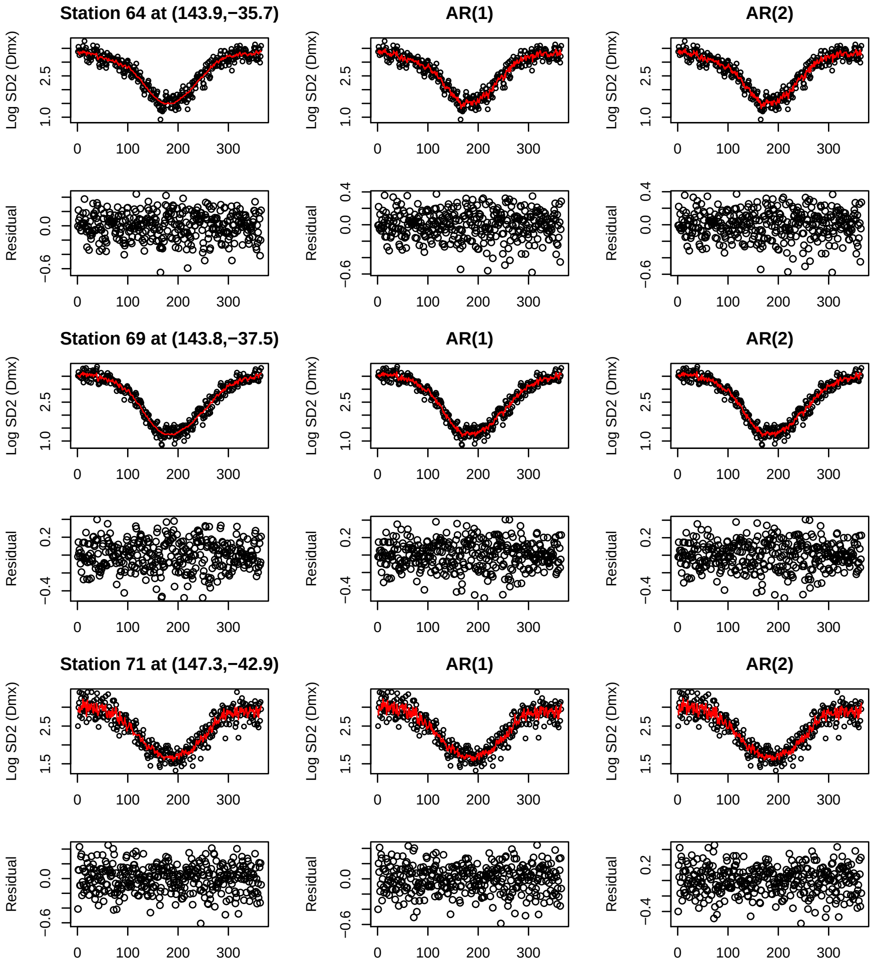

Figure 5: Fitting results of inter-annual variance with model 3, 4 and 5 under loagrithm transformation.

S3. Simulation Study

Let $L = 4$, then $\kappa_1 = 0, \kappa_2 = 0.25, \kappa_3 = 0.5, \kappa_4 = 0.75, \kappa_5 = 1$. Let $y_t(\mathbf{s})$ be the simulated data point of day t and location $\mathbf{s}$, and $y_t(\mathbf{s})$ follows a piece-wise normal distribution with pdf f.

$$f(y_t(\mathbf{s})) = \sum_{l=1}^{L} I_{\{q(\kappa_l) \leq y_t(\mathbf{s}) \leq q(\kappa_{l+1})\}} N({}_t(\mathbf{s})|a_l(\mathbf{s},t), b_l(\mathbf{s},t)^2),$$

where $b_l(\mathbf{s},t) = \theta_{0l}(\mathbf{s}) + t\theta_{1l}(\mathbf{s})$ and $a_l(\mathbf{s},t) = q(\kappa_{l+1}|\mathbf{s},t) - b_l(\mathbf{s},t)\Phi^{-1}(\kappa_{l+1})$ if $\kappa_l < 0.5$ and $a_l(\mathbf{s},t) = q(\kappa_l|\mathbf{s},t) - b_l(\mathbf{s},t)\Phi^{-1}(\kappa_l)$ if $\kappa_l \geq 0.5$. The generated

$\{y_t(\mathbf{s})\}$ need to be corrected so that the variance can have the similar feature in real series. The procedure to generate $y_t(\mathbf{s})$ is as shown in Procedure 1.

Procedure 1 Generation of simulated data

1. Generate $X_t(\mathbf{s}) \sim N(0,1)$, $t = 1, \dots, T$.
2. Set $y_t^*(\mathbf{s}) = \sum_{l=1}^4 I_{\{\Phi^{-1}(\kappa_l) \le X_t(\mathbf{s}) \le \Phi^{-1}(\kappa_{l+1})\}} X_t(\mathbf{s}) b_l(\mathbf{s}, t) + a_l(\mathbf{s}, t)$ for all t.
3. Let

$$y_t(\mathbf{s}) = \frac{y_t^*(\mathbf{s}) - \hat{u}_d(\mathbf{s})}{\hat{\sigma}_d(\mathbf{s})} \sigma_t(\mathbf{s}) + \hat{u}_d(\mathbf{s}),$$

where t is dth day of a year, and $\hat{u}_d(\mathbf{s})$ and $\hat{\sigma}_d^2(\mathbf{s})$ are sample mean and variance of generated $y_t^*(\mathbf{s})$.

To simulate the data with features more close to real temperature series, we select 10 stations and do a pilot run of the quantile regression to get their corresponding quantile function $q(\kappa|\mathbf{s}, t)$ for $\kappa = 0.25, 0.5$ and 0.75. Then we generate the temperature series according to these quantile functions using the procedures outlined above. The simulated series have the same heterogeneity as the real data.

With these simulated data, we run the models Eq. (10) and Eq. (11) in main text separately. The true and estimated values (over 100 simulations) of trend function are shown in TABLE 3. The column RMSE indicates the root mean squared values of difference between true and estimated values. Total RMSE shows the summation of RMSE over all stations and three quantiles. The column $\frac{RMSE(\text{no } \sigma_t)}{RMSE(\sigma_t)}$ shows the ratio between RMSE of estimated values from model without and with σ_t. For $\frac{RMSE(\text{no } \sigma_t)}{RMSE(\sigma_t)} > 1$ it means that including σ_t as a covariate improves the model in terms of RMSE. As shown in Table 3, for most rows (18 of 30), the model including σ_t outperforms the model without σ_t, but there are 5 rows for which including σ_t reduces the performance. In term of total squared error, model with $\sigma_t(\mathbf{s})$ as a co-variate outperforms the model without $\sigma_t(\mathbf{s})$. Hence, we conclude that model Eq. (11) in general can detect the true trend more accurately.

Table 3: Comparison of results (estimated values of trend function) with and without including σ_t as a covariate

Station	τ	True trend	Trend (no σ_t)	RMSE	Trend (σ_t)	RMSE	$\frac{RMSE(\text{no } \sigma_t)}{RMSE(\sigma_t)}$
1	tau=0.25	0.43	0.51	0.09	0.53	0.11	0.86
	tau=0.5	0.59	0.58	0.06	0.6	0.03	1.75
	tau=0.75	0.49	0.55	0.08	0.57	0.08	0.96
2	tau=0.25	1.03	0.91	0.15	0.97	0.07	2.14
	tau=0.5	0.80	0.73	0.11	0.76	0.06	1.69
	tau=0.75	0.49	0.57	0.10	0.61	0.12	0.83
3	tau=0.25	1.12	1.19	0.14	1.17	0.08	1.76
	tau=0.5	0.91	1.02	0.16	0.97	0.10	1.68
	tau=0.75	0.68	0.81	0.15	0.82	0.14	1.04
4	tau=0.25	0.14	0.14	0.02	0.19	0.05	**0.37**
	tau=0.5	0.22	0.26	0.06	0.29	0.07	**0.78**
	tau=0.75	0.26	0.27	0.03	0.31	0.05	**0.63**
5	tau=0.25	1.01	1.11	0.14	1.16	0.15	0.96
	tau=0.5	0.77	0.83	0.09	0.83	0.07	1.38
	tau=0.75	0.96	0.92	0.09	0.93	0.03	2.86
6	tau=0.25	1.05	0.98	0.11	1.05	0.02	4.97
	tau=0.5	1.94	1.29	0.66	1.4	0.55	1.21
	tau=0.75	1.12	0.85	0.28	0.9	0.22	1.29
7	tau=0.25	-1.90	-1.75	0.21	-1.87	0.05	4.61
	tau=0.5	-1.42	-1.31	0.16	-1.3	0.13	1.25
	tau=0.75	-0.86	-0.95	0.12	-0.93	0.08	1.52
8	tau=0.25	-0.47	-0.54	0.09	-0.48	0.04	2.13
	tau=0.5	0.46	0.22	0.24	0.38	0.10	2.53
	tau=0.75	-0.03	-0.13	0.11	-0.07	0.06	1.95
9	tau=0.25	-0.05	0.08	0.15	0.19	0.27	**0.56**
	tau=0.5	0.40	0.64	0.27	0.66	0.30	0.91
	tau=0.75	0.65	0.83	0.20	0.78	0.14	1.44
10	tau=0.25	0.14	0.21	0.10	0.26	0.15	**0.64**
	tau=0.5	0.07	0.38	0.35	0.38	0.36	0.99
	tau=0.75	0.32	0.46	0.18	0.48	0.20	0.90
Total RMSE				4.71		3.86	

S4. Result of Quantile Trend by Season

This section shows the quantile trend for summer and winter seasons. Figure 6 shows the results for summer for three quantile 0.1, 0.5 and 0.9. Under all three quantiles,the south-east part of Australia consistently has warming summer, including NSW, VIC, SA, TAS and south QLD, where most stations present a large increase $\geq 0.3°$C per decade in last 60 years. In contrast, the south-west region shows no significant trend of temperature for summer, with per decade increase $\leq 0.1°$C for most stations in the region. The northern part of Australia

generally has no significant trend of warming for most stations.

In terms of Dmn, stations in coastal area of NSW and inland area of QLD show an increase by $\geq 0.2°C$ for 0.1 quantile, and stations in inland areas of QLD for 0.5 and 0.9 quantiles. Generally in summer, daily maximum temperatures (of both hot and cold days) get much hotter in the south-east regions, while they get only slightly hotter in other regions. For daily minimum temperature, days get much warmer in south QLD in general. And cold days get much warmer in the NSW coastal region. Other regions experience a relatively smaller warming trend with most stations showing an increase $\leq 0.2°C$ and even decrease.

As for winter temperature in Figure 7, we can see from the Dmx series that the south part of Australia experiences less increase than the north part (QLD) except TAS having a station with substantial increase ≥ 0.4 under all three quantiles. Within QLD, the southern area shows an increasing trend $\geq 0.2°C$ that is more than the far north area where one station shows a cooling trend. The Dmn series also show that the northern area (especially QLD) increases more than southern region, but the difference is that the southern area (especially VIC, NSW, TAS and south part of WA) shows a clear cooling trend under all three quantiles, though quantile 0.9 decreases less than quantile 0.1 and 0.5. In QLD, generally higher quantiles increase less than lower quantiles, e.g., for quantile 0.1, most stations show an increase with $\geq 0.3°C$, while for quantile 0.9 the increase amounts are mostly in 0.2°C to 0.3°C. One station in far north QLD shows non-significant increase or even decrease in these quantiles.

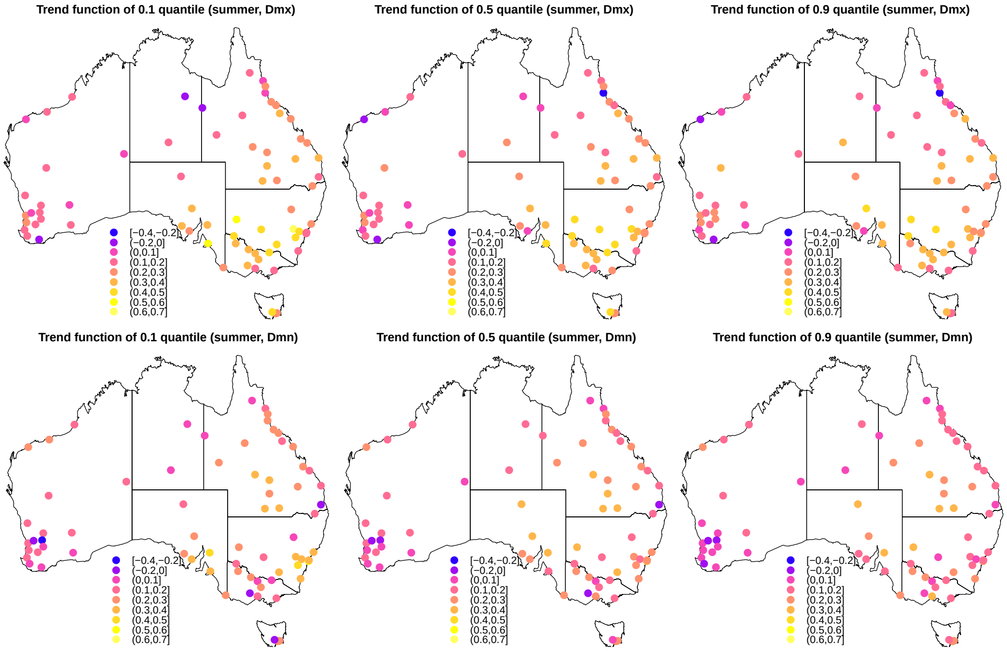

Figure 6: Values of trend function for $\tau = 0.1, 0.5$ and 0.9 for Dmx (top row) and Dmn (bottom) in summer (December, January and February).

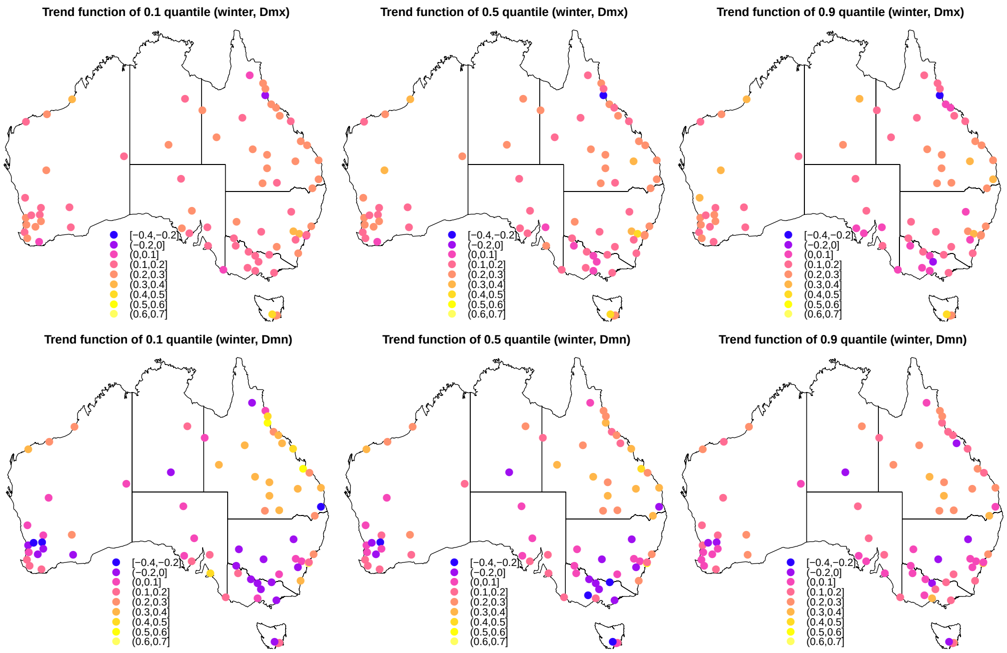

Figure 7: Values of trend function for $\tau = 0.1, 0.5$ and 0.9 for Dmx (top row) and Dmn (bottom) in winter (June, July and August).

References

[1] Fred Espen Benth and Jūratė Šaltytė Benth. Stochastic modelling of temperature variations with a view towards weather derivatives. *Applied Mathematical Finance*, 12(1):53–85, 2005.

[2] Fred Espen Benth and Jūratė Šaltytė Benth. The volatility of temperature and pricing of weather derivatives. *Quantitative Finance*, 7(5):553–561, 2007.

[3] Jūratė Šaltytė Benth, Fred Espen Benth, and Paulius Jalinskas. A spatial-temporal model for temperature with seasonal variance. *Journal of Applied Statistics*, 34(7):823–841, 2007.

[4] Sean D Campbell and Francis X Diebold. Weather forecasting for weather derivatives. *Journal of the American Statistical Association*, 100(469):6–16, 2005.

[5] Heather Mitchell, Michael D McKenzie, et al. Garch model selection criteria. *Quantitative Finance*, 3(4):262–284, 2003.

[6] B Sirangelo, T Caloiero, R Coscarelli, and E Ferrari. A stochastic model for the analysis of maximum daily temperature. *Theoretical and Applied Climatology*, 130(1-2):275–289, 2017.
