# Supplementary material for: Spatio-temporal quantile regression analysis revealing more nuanced patterns of climate change: A study of long-term daily temperature in Australia
Source: PLoS One. 2022 Aug 24;17(8):e0271457. doi: 10.1371/journal.pone.0271457 (PMC9401128; doi:10.1371/journal.pone.0271457)
Supplement: S2 File — (ZIP) [file pone.0271457.s002.zip › sr_submission.pdf]

Spatio-temporal quantile regression analysis revealing more nuanced patterns of climate change: a study of long-term daily temperature in Australia

Qibin Duan[1], Clare A. McGrory[1], Glenn Brown[1], Kerrie Mengersen[1], and You-Gan Wang[2,1,*]

[1]School of Mathematical Sciences, Queensland University of Technology, Australia
[2]Institute for Learning Sciences & Teacher Education, Australian Catholic University, Brisbane 4000, Australia
[*]you-gan.wang@acu.edu.au

ABSTRACT

Climate change is commonly associated with an overall increase in mean temperature in a defined past time period. Many studies consider temperature trends at the global scale, but the literature is lacking in in-depth analysis of the temperature trends across Australia in recent decades. In addition to heterogeneity in mean and median values, daily Australia temperature data suffers from quasi-periodic heterogeneity in variance. However, this issue has barely been overlooked in climate research. A contribution of this article is that we propose a joint model of quantile regression and variability. By accounting appropriately for the heterogeneity in these types of data, our analysis reveals that daily maximum temperature is warming by $\sim 0.21°$C per decade and daily minimum temperature by $\sim 0.13°$C per decade. However, our modeling also shows nuanced patterns of climate change depends on location, season, and the percentiles of the temperature series over Australia.

Introduction

The increase in the intensity and frequency of global extreme weather events, e.g., extreme heat, extreme cold, drought, snow cover decline, is attributed to fundamental changes in the underlying climate[1,2]. There is also an overall warming trend occurring with the global mean temperature estimated to have risen by 0.85°C during 1880-2012[3]. This increasing trend is predicted to continue. These changes have already caused and will continue to induce substantial societal and ecological impacts. Unchecked, climate change is hence a major threat now faced by the entire world . Human activity is widely argued to be a major cause of global warming[4,5], but a great deal of uncertainty still remains regarding the exact mechanisms that underlie the warming process[6]. As the study[6] highlight, the global surface warming of recent decades has been realized as a succession of periods of warming slowdowns, or hiatus, followed by warming surges.

While warming is the trend globally, at the regional and local scales, a wide variety of changes in temperature is observable across the globe[7,8]. Climate changes have been extensively studied at the global scale, but there is also intense interest in understanding patterns of change at a national level[9]. However, this work is still emerging. Within Australia, for example, in-depth statistical studies on historic temperature changes are limited[10]. Australia is the largest country in Oceania and one of the major producers of agricultural products, so deep understanding of climate change in Australia is of importance.

According to the State of the Climate[11], Australia's climate has warmed on average by $1.44 \pm 0.24°$C since 1910, with most of the warming having occurred since 1950. This has been accompanied by increased frequency of extreme heat events. As reported in more detail in the Climate Statement, the overall mean (average of daily maximum and minimum) temperature for the 10 year period from 2010 to 2019 was the highest on record, at 0.86°C above the long term overall average since 1910, and 0.31°C warmer than the 10 years 2000–2009, which is the second warmest 10-year period[12]. The climate change within Australia is also spatially varied. For example, for the wet tropical area in far North Queensland, the mean temperature increased by 1.1°C between 1910 and 2013 using a linear trend, while in the east coast area in New South Wales, the mean temperature increased by 0.8°C in the same period. Moreover, for the Rangelands area, the north part increased 1°C and the south part increased 0.9°C during the same period[13].

The majority of statistical analyses undertaken in climate studies in Australia are based on inference about the maximum, minimum or mean temperature. However, a focus on these measure alone can fail to detect more nuanced patterns of change over space and time. In this article we take an approach based on quantile regression estimates of the data with consideration of spatial correlation. Quantile regression was originally proposed by Koenker and Bassett (1978) as an alternative approach to mean regression that does not require the usual strict assumption about normally distributed residuals in the regression models[14]. The idea has been used to identify changes over time of any percentiles of climate variables; see[15–18] for particular focus on

the analysis of temperature series. The performance of quantile regression for trend detection analysis has been favorably compared with traditional approaches, such as robust linear regression and the nonparametric M-K test[17]. The spatial and temporal variation can be modeled jointly as in Reich (2012), in a Bayesian manner[19].

One advantage of this approach is that quantile regression estimates are less influenced by extreme outliers in the response measurements than standard linear regression estimates based on the mean. The other big motivation for this approach is that the conditional quantile functions are of interest to us. The variety of measures of central tendency and statistical dispersion allow us to describe the relationship at different points in the conditional distribution of the outcome. In this way we obtain a more comprehensive picture of the relationship between the variables. When using quantile regression, we have increased freedom in our modelling in that we are not restricted by the assumption that variable relationships are the same at the median and tails of the distribution as they are at the mean. Such models could provide a more in-depth understanding of the historic change in Australian temperature, which can potentially improve anticipation and management of climate risk and associated negative impacts.

We highlight however, that in analysis of daily Australia temperature data, complicated heterogeneity in variance arises and this must be addressed if results are to be reliable. In this article we propose a joint model including variance as a covariate in our spatio-temporal regression to do so.

Results

In this study we analyze the spatio-temporal pattern of warming in Australia based on the trends of averages and quantiles of daily maximum and minimum temperatures using recent observed data. The trend function of different quantiles are obtained with using the joint model of quantile regression and variability presented in "Methods" section

Data collection and selection

The geographical boundary of Australia is between $9° - 44° S$ latitude and $112° - 154° E$ longitude (apart from Macquarie Island), with total area of $7,692,024$ km^2. The massive size of the country gives it a wide variety of landscapes, with tropical rainforests in the north-east, mountain ranges in the south-east, south-west and east, and desert and semi-arid land in the center, resulting in a variety of climates across the country. The northern part of the country has a tropical climate, with predominantly summer rainfall. As for the southern part, the south-west corner of the country has a Mediterranean climate, while the south-east ranges from oceanic (Tasmania and coastal Victoria) to humid subtropical (from the upper half of New South Wales), with highlands featuring alpine and subpolar oceanic climates. The interior desert has stable arid and semi-arid climates. More details about the Australian climate can be found on the website of Bureau of Meteorology (BoM), Australia[20].

The daily maximum (Dmx) and minimum (Dmn) temperature series were obtained from 1,745 weather stations operated by the BoM Australia. These datasets can be accessed through the `R` package `bomrang`[21]. The length of these time series varies among different stations from less than 10 years to more than 100 years of observations. The latitude, longitude and elevations of each station are also obtained from the BoM website.

We restricted the study period from Jan 1, 1960 to Dec 31, 2019, so that most warming periods can be covered and more operating stations could be included[22]. As the years of 2000 and 2010 are the starting years of the top two warmest 10-year periods in Australia on record, we considered data from stations not covering these periods to be less meaningful in terms of studying the warming trend in Australia. Therefore, we excluded stations that were not operating during these periods. Furthermore, we excluded the stations with over 20% of missing observations. As a result, data from 72 stations were included in our analysis. These stations are geographically distributed as shown in FIG 1. Specifically, there are 22 stations in Queensland (QLD), 18 in Western Australia (WA), 11 in New south Wales (NSW), 9 in Victoria (VIC), 7 South Australia (SA), 4 in Tasmania (TAS) and 2 in the Northern Territory (NT). The ID number of each station is also included for the sake of description of results.

Some examples of pronounced societal and ecological effects associated with variations in Australia temperatures are as follows:

- A heatwave occurs when the daily maximum and minimum temperatures are unusually hot over a three-day period at a location. Heat waves affect human health, transportation infrastructure, electricity demand and infrastructure performance, outside labor productivity, and ecology and are a risk to natural systems (e.g., bush fires).
- Duration of elevated temperatures affects human health, outside labour productivity, transport infrastructure, electricity demand etc. For instance, CSIRO research shows that 4 sequential days of elevated temperature at 35°C requires 33% more cooling energy for a building than does 4 distributed days at the same temperature.
- Daily and seasonal temperature ranges affect agricultural productivity in terms of crop yields and protein content, and transportation infrastructure.

- Rate of change of warming impacts on human health and ecological communities by reducing the time available to adapt to changing conditions.

- Anomalous and unpredictable temperature patterns increase the risk of adverse events such as bush fires.

Trend analysis based on quantiles

FIG 2 shows the values of the trend functions for quantiles 0.1, 0.5 and 0.9 for each of the included stations, respectively, while FIG 3 and 4 show the change in trend function with quantile levels for stations with any trend function > 0.3°C . Note that the value represents the total increases in degrees Celsius per decade during the study period of 60 years. For daily maximum temperature, the average warming trend of all 72 stations is 0.22, 0.22 and 0.20°C per decade for quantile levels 0.1, 0.5 and 0.9, respectively, while these values become 0.14, 0.13, 0.14°C for daily minimum temperature.

For all three quantile levels, the daily maximum tempertures are increasing in all stations except for one in north Queensland (station 31 in FIG 1 located in Cardwell Marine Pde QLD), and the values of the trend function per decade mostly lie in the range $(0.1, 0.3)$ indicating a warming trend of 0.6°C to 1.8°C in total during the last 60 years. For quantile 0.1, representing cold daily maximum temperture, there are five stations in NSW that increase by more than 0.3°C per decade during the study period. Three of these (stations 50, 59 60) are within the range $(0.3, 0.4)$ and decrease slightly to $(0.2, 0.3)$ for larger quantiles, and another two (station 52, 53) exceed 0.4 and also decrease for higher quantiles but remain > 0.2 (FIG 4). Moreover, other stations (station 38 and 48 in QLD, 8 in WA, 26 in SA, 72 in TAS) have been warming ≥ 0.3°C for all three quantile levels per decade, especially for station 8 in TAS around 0.4°C. Station 61 in VIC has been warming > 0.3°C for the 0.1 quantile, but this decreases to < 0.3 for higher quantiles. For quantile 0.5, representing median daily maximum temperture, there are eight stations having > 0.3°C increase per decade. Of these, stations 20 in NT and 35 in QLD have smaller increases in the 0.1 quantile < 0.3°C (FIG 3), and three have > 0.4°C increase with two in NSW (station 52 and 53) and one (station 72) in TAS. In terms of quantile 0.9, for hot daily maximum temperture, six stations show the > 0.3°C increase with two in QLD (station 38 and 48), and one in NSW (station 55), TAS (station 72), WA (station 4) and NT (station 20). The station 31 in QLD (located in Cardwell Marine Pde) shows a declining trend under both 0.5 and 0.9 quantiles (the top panels of FIG 2).

The pattern of the change in trend of Dmn is different from Dmx, as shown in the bottom panels of FIG 2, where the values of the trend functions per decade mostly lie in the range $(0.0, 0.3)$, and more stations have a cooling trend with negative values for trend functions. The trend function of the quantile 0.1 represents the change of temperature for the extremely cold days, for which there are 12 stations that show a cooling trend and six of them have been cooling > 0.1°C per decade during the study period, i.e., station 12 and 13 in WA, 44 in QLD, 65 and 69 in VIC, and 72 in TAS. Nine stations show an increasing trend with amount > 0.3°C per decade, i.e., station 3 in WA, station 26 in SA, six stations in QLD (station 31,34,36,39,41 and 42), and station 55 in NSW (> 0.4°C). For the quantile level 0.5, eight stations show a cooling trend in the south east and south west of Australia with negative values for the trend function, and four (stations 12, 44, 65 and 69) have been cooling > 0.1°C per decade. Three stations (stations 3, 39 and 55) in WA, NSW show a warming trend with amount > 0.3°C. For the quantile level 0.9, only four stations still show a cooling trend with two (stations 12 and 13) in WA, one (60) in NSW and one (63) in VIC. Three stations have an increasing trend with > 0.3°C degrees with one (station 3) in WA, two (stations 29 and 39) in QLD and one in NSW (station 67).

In terms of the trend function against quantile levels, FIG 3 and 4 display stations with any trend function > 0.3°C. Note that red curve above blue curve indicates that Dmx has increased more than Dmn, and vice versa. If trend values increase with quantile level, it indicates bigger variation in the series, and vice versa. A positive trend value in high quantile of Dmx means more extreme heat events and a negative value of trend function in low quantile of Dmn means more extreme cold events. For example, stations 4, 8, 20, 35 and 38 in FIG 3 and stations 50, 52, 53, 59, 60, 61 and 72 in FIG 4 present such a pattern with red curve above blue curve. For station 4, larger quantiles increase more than smaller quantiles for both Dmx and Dmn with all > 0, indicating warming trend and bigger variation in both series, and more extreme heat events and less extreme cold events. Station 8 has a pattern with warming trend but smaller variation in both series. While station 20 shows a warming trend in Dmx series with bigger variation and more extreme heat events. As for station 35, it presents a warming trend with more extreme heat events but no bigger variation in Dmx. Station 52 and 53 present a similar pattern with trend coefficient decrease with quantile level in both series, which indicates a warming trend but smaller variation. Station 60 and 61 have a warming trend in Dmx, but no clear trend in Dmn. Stations 50 and 59 have a warming trend in both series with smaller variation in Dmx. Station 3, 29, 31, and 39 FIG 3 and station 41 in FIG 4 have the blue curve above the red curve. Especially for station 31, the Dmx series present a cooling trend for trend coefficient of most quantile < 0 and a smaller variation. The Dmn show a warming trend and a smaller variation. Although there are clear trends in both Dmx and Dmn, when it comes to the mean temperature, such trends might be difficult to obtain. For this station, the extreme events occur less.

Note that some stations have red and blue curves crossed, e.g., station 36, 42, 55 and 67, where at that quanitle level both Dmx and Dmn have same trend.

Spatial pattern of trend

The spatial pattern of trend functions across Australia is shown in Figure 5 for the trend of Dmx (in the top panels) and Dmn (in the bottom panels), respectively. For 0.1 quantile of Dmx, the values of trend function are all positive with warming trend for cold days (after removing seasonality). The north-east area including NSW, VIC, TAS, south part of QLD and east part of SA appear to experience more of an increase than other regions under the 0.1 quantile of Dmx, with ≥ 0.3°C increase per decade in the last 60 years. This area covers the most part of the Murray–Darling basin, which experienced water loss in the last few years[23]. We especially highlight TAS and greater Sydney area which increase the most at nearly 0.4°C. In contrast, the far north QLD and the north-west corner of WA have the least change with around 0.1°C increase in the same period. In terms of the 0.5 quantile of Dmx, TAS and parts of NSW still have mostly increasing temperatures with > 0.3°C, while other areas have 0.1°C to 0.3°C increase, except for the north of QLD and the north-west corner of WA showing a non-significant increase ≤ 0.1°C. When it comes to the 0.9 quantile, parts of the north of QLD show a non-significant increase, or even decrease. Other regions generally have a warming trend of 0.2°C to 0.3°C.

The trend of Dmn series perform differently. For 0.1 quantile of Dmn, south QLD, small region of north coast of WA, coastal area near Sydney and Adelaide show a warming trend around ≥ 0.3°C. These areas continue to show warming trend for the 0.5 quantile, but not for the 0.9 quantile. While other regions have less of an increase with ≤ 0.2°C for 0.1 quantile, and some areas (south-east and south-west region of Australia) even show a cooling trend with a negative value of trend function. For 0.5 quantile, most parts of Australia does not show a significant increase, with most values of trend function lying 0 to 0.1°C, while the south-east area experiences a consistent cooling. However, no significant cooling trend is shown for the 0.9 quantile. Instead, inland parts of QLD and north coast of WA show an increase with 0.3°C.

Discussion

This article has provided a detailed looked at the changes in temperature across Australia in recent decades.

In this study, we raised the heterogeneity issue in the daily temperature time series with an exploratory analysis, where the intra-annual sample variance has quasi-periodic variations and temporal correlations. Such issues lead to inaccurate estimation of parameters coefficients, if they are not handled appropriately. Specifically, the trend detected might be misleading to a large extent. Many studies also suggest that the Long-range dependence (LRD) in a time series, if a time series displays a slowly declining autocorrelation function (ACF) in variance, can significantly increase the uncertainty of trend detection[17,24–27]. Note that LRD can be also reflected from the ACF of residuals in a mean regression model. To include all the heterogeneity of variance into the model, GARCH model is used to model the variance of temperature time series. It also suggests that GARCH model can capture the shape of the ACF of volatility in daily financial return series, and is consistent with long-memory based on semiparametric and parametric estimates[28]. Hence, our variance model not only accounts for seasonality and temporal correlation, but also reduces the uncertainty caused by LRD.

Spatio-temporal quantile regression has been applied to analyze the temperature series from 72 meteorological stations in Australia. By including the variance term as co-variate in the quantile model, the proposed quantile regression approach has considered all heterogeneity including quasi-periodic variations and temporal correlations. We only include the linear trend in the modeling as both linear and quadratic trend showed a similar pattern during the study period. Hence, our model can detect a reliable trend in the temperature time series.

With analysis of results, we confirm the different patterns of climate change for different percentiles of daily maximum and minimum temperature series over Australia. Overall, the country appears to be experiencing a warming in daily maximum temperature except for a small area in far north QLD, and both cold and hot days are tending to get get warmer. A warming trend in hot days will lead to more frequent extreme heat events. Generally, NSW, south QLD and TAS experience the most significant warming in daily maximum temperature compared with other regions.

In terms of daily minimum temperature, South QLD and the north coast of WA experience more warming than other part of Australia. However, the warming trend is less significant than for daily maximum temperature. The daily minimum temperature series is associated with extreme cold events. Specifically, VIC, TAS and south WA have increasing number of extreme cold days. Notably, South QLD experiences an overall climate warming in both maximum and minimum temperature, leading to more frequent hot days and fewer cold days. The year round warming trends in South QLD may have significant adverse impacts on agricultural production in Queensland which is currently aiming to boost from AUD$60 to 100 billion by 2030. NSW experiences a climate warming in daily maximum temperature with more hot days, but stable daily minimum temperature. VIC and TAS get a more extreme weather, where VIC get much more extreme cold days and slightly more extreme hot days, while TAS has much more extreme hot days and slightly more extreme cold days. When we look into summer, NSW, VIC, SA and south QLD experience a more warming summer than other regions that have no significant warming trend in summer. Winter generally gets warmer in most regions in Australia but gets less warmer in VIC with regards to daily maximum temperature. In

daily minimum temperature series, it gets quite a bit warmer in QLD, while other regions only get slightly warmer or even colder in south WA, NSW and VIC.

It is worthy nothing that our model results in different changing rates with that presented in regional reports[13,29–34]. This is because these reports focused on the period between 1910 and 2013 with using a linear trend to obtain the changing rate. However, as noted in the report[35], most warming in Australia occurs after 1950 and post-1950 period has faster warming rate than pre-1950 period.

There are some limitations we need to take into account when interpreting the results and analysis. The included stations are not distributed evenly as there are very few stations in the inland region of WA, NT and SA. This could lead to over-generalization of conclusions in these areas. Also, in spatio-temporal quantile regression, we only consider the location coordinates of stations, but we do not to account for the impact of geography, e.g., river catchment, mountain range and distance to ocean.

Methods

Exploratory Analysis

The daily temperature time series are affected by seasonality, with quasi-periodic variations in both sample mean and variance; see[36–39]. An example is shown in the bottom panels in Fig 6, where the sample mean and variance of a particular station are calculated over 60 years of observations. Other stations had different patterns, depending on the respective ecosystem, but they similarly exhibited this phenomenon.

Such mean periodicity in the series can be easily handled with parametric harmonic functions (or truncated Fourier series). Ignoring the seasonality or heterogeneity in variance for the time being, the observed daily temperature time series can be modelled as follows.

$$y_t(\mathbf{s}) = \mu_t(\mathbf{s}) + \sigma(\mathbf{s})\epsilon_t(\mathbf{s}), \quad \epsilon_t(\mathbf{s}) \sim_{iid} N(0,1), \tag{1}$$

where t is t-th day from January 1, 1960 to December 31, 2019, $\mathbf{s}$ is a particular station, and expected value $\mu_t(\mathbf{s})$ is given by

$$\mu_t(\mathbf{s}) = \beta_0(\mathbf{s}) + \beta_1(\mathbf{s})t + \beta_2(\mathbf{s})\mathbf{x}_t(\mathbf{s}) + FSk(t, \boldsymbol{a}(\mathbf{s}), \boldsymbol{b}(\mathbf{s})) + \sum_{i=1}^{p} \rho_i(\mathbf{s}) e_{t-i}(\mathbf{s}). \tag{2}$$

Note that $\epsilon_t(\mathbf{s})$ is assumed white-noise and small-scaled relative to the scale of $\mu_t(\mathbf{s})$ and $\sigma^2(\mathbf{s})$ is constant in location $\mathbf{s}$. Here $FSk(t, \boldsymbol{a}(\mathbf{s}), \boldsymbol{b}(\mathbf{s}))$ is the k-th order truncated Fourier series, and $\sum_{i=1}^{p} \rho_i(\mathbf{s}) e_{t-i}(\mathbf{s})$ is an $AR(p)$ process.The term $\beta_2(\mathbf{s})\mathbf{x}_t(\mathbf{s})$ is the term for other covariates, and in the following we consider the inclusion of the Southern Oscillation Index (SOI) values. For simplicity of notation, we denote the kth order truncated Fourier series as follows

$$FSk(t, \boldsymbol{a}(\mathbf{s}), \boldsymbol{b}(\mathbf{s})) = \sum_{j=1}^{k} \left[a_j(\mathbf{s}) \sin\left(\frac{2\pi jt}{D_t}\right) + b_j(\mathbf{s}) \cos\left(\frac{2\pi jt}{D_t}\right) \right],$$

where $\boldsymbol{a}(\mathbf{s}) = (a_1(\mathbf{s}), \dots, a_k(\mathbf{s}))$ and $\boldsymbol{b}(\mathbf{s}) = (b_1(\mathbf{s}), \dots, b_k(\mathbf{s}))$ are the coefficients of harmonic terms for station $\mathbf{s}$, and D_t denotes the number of days in the respective year.

The red curve in the top left panel of Fig 6 shows the fitted values of $\mu_t(\mathbf{s})$ for one station for illustration; these are obtained from the model in Eq (1). The top right panel shows the residuals against fitted values which clearly indicates the heterogeneity issue in variance. This leads to the failure of the equal variance assumption in Eq (1). Tests of of temporal auto-correlation are shown in the middle panels: for this station, no substantial auto-correlation is found for the residuals, while significant and quasi-periodic auto-correlation is found in the squared residuals.

Although the heterogeneity issue is not emphasized and indeed often ignored in many climate themed articles, some models have been proposed to address it. For instance, in the model of the daily average temperature of four US cites, variance is accounted for via a GARCH process[36]. In some studies, for example, ARCH models were employed[37,38,40], while Sirangelo (2017) models the inter-annual sample variance $\hat{\sigma}_d^2(\mathbf{s})$ as simple periodic functions[39].

In this study, we model the variance of daily temperature with the inter-annual sample variance. Let $y_{i,d}(\mathbf{s})$ be the maximum (or minimum) daily temperature on the d-th day of year i recorded at station $\mathbf{s}$. Note that we assume there are 365 days for each year and February the 29th was omitted in the analysis.

For each particular day, d, for station $\mathbf{s}$, we compute the inter-annual sample mean and variance, denoted as $\hat{\mu}_d(\mathbf{s})$ and $\hat{\sigma}_d^2(\mathbf{s})$:

$$\hat{\mu}_d(\mathbf{s}) = \frac{1}{T}\sum_{i=1}^{T} y_{i,d}(\mathbf{s}), \qquad \hat{\sigma}_d^2(\mathbf{s}) = \frac{\sum_{i=1}^{T}(y_{i,d}(\mathbf{s}) - \hat{\mu}_d(\mathbf{s}))^2}{T-1}, \tag{3}$$

where T is number of years that are included in the analysis.

The inter-annual mean is modeled as follows

$$\mu_d(\mathbf{s}) = \hat{\mu}_d(\mathbf{s}) + \epsilon_d(\mathbf{s}), \tag{4}$$

where $\epsilon_d(\mathbf{s}) = \hat{\sigma}_d(\mathbf{s})\delta_d(\mathbf{s})$ and $\delta_d(\mathbf{s}) \sim_{iid} N(0,1)$. We include the inter-annual mean and squared mean as a covariate of variance function at the end of the equation below. The model selection results can be found in Supplementary document Section S1.

$$\log\left(\sigma_d^2(\mathbf{s})\right) = \beta_0(\mathbf{s}) + \beta_1(\mathbf{s})\mu_d(\mathbf{s}) + \beta_2(\mathbf{s})\mu_d^2(\mathbf{s}) + FSk(d, \boldsymbol{a}(\mathbf{s}), \boldsymbol{b}(\mathbf{s})) + \rho_1(\mathbf{s})\left(\hat{\sigma}_{d-1}^2(\mathbf{s}) - \sigma_{d-1}^2(\mathbf{s})\right). \tag{5}$$

We have tested and compared a number of variance models to account for the inter-annual heterogeneity that varies from site to site. Different orders of Fourier series have been tested and selected as $k = 4$. Quantile regression requires the heterogeneity function at each quantile level (except $\tau = 0.50$), we therefore need to jointly estimate the regression parameters and variance parameters.

Joint Models for Quantile Regression and Variability

Quantile regression permits simultaneous analysis of several features of the response distribution. To jointly model all quantiles simultaneously and spatially, while accounting for heterogeneity, we propose here an improved version of the spatio-temporal quantile regression approach proposed by Reich (2012) and apply it to the Australian daily temperature data[19].

Now considering heterogeneity in variance, the model in Eq (1) can be modified as follows:

$$y_t(\mathbf{s}) = \mu_t(\mathbf{s}) + \sigma_t(\mathbf{s})\epsilon_t(\mathbf{s}), \quad \epsilon_t(\mathbf{s}) \sim_{iid} f(\mathbf{s}), \tag{6}$$

where $f(\mathbf{s})$ is the PDF of $\epsilon_t(\mathbf{s})$; we denote $F(\mathbf{s}) \in [0,1]$ to be the corresponding CDF at location $\mathbf{s}$.

The quantile function $q(\tau|\mathbf{s},t)$ is the function that satisfies $\mathbb{P}\{y_t(\mathbf{s}) < q(\tau|\mathbf{s},t)\} = \tau \in [0,1]$. Inserting Eq (6) into this expression we have $\mathbb{P}\{\mu_t(\mathbf{s}) + \sigma_t(\mathbf{s})\epsilon_t(\mathbf{s}) < q(\tau|\mathbf{s},t)\} = \tau$ and the quantile function $q(\tau|\mathbf{s},t)$ can be expressed as follows.

$$q(\tau|\mathbf{s},t) = \mu_t(\mathbf{s}) + \sigma_t(\mathbf{s})F^{-1}(\tau), \tag{7}$$

where $F^{-1}(\tau)$ is the inverse CDF. If the error term $\epsilon_t(\mathbf{s})$ is assumed normally distributed, then $F^{-1}(\tau) = \Phi^{-1}(\tau)$. In this study, we are interested in testing the changes in the quantile function $q(\tau|\mathbf{s},t)$ over time t for each τ. Therefore, in Eq (7), the term $\sigma_t(\mathbf{s})$ needs to account for the time variable t. For example, for the case of a Gaussian distributed response with linear trend over time in Chandler (2005) , the mean is modeled as $\mu_t(\mathbf{s}) = \beta_0(\mathbf{s}) + \beta_1(\mathbf{s})t$, and the standard deviation $\sigma_t(\mathbf{s}) = \theta_0(\mathbf{s}) + \theta_1(\mathbf{s})t$, with $F^{-1}(\tau) = \Phi^{-1}(\tau)$[41]. Here, the standard deviation changes linearly with time t. The Gaussian assumption is generalized by Reich (2012), by incorporating a piece-wise Gaussian basis function to describe the linearly changing heterogeneity more flexibly for a non-Gaussian distributed response[19]. Furthermore, this model is able to characterize the entire quantile process with accommodation of non-Gaussian features, such as asymmetry and heavy or light tails. However, the model of Reich (2012) was proposed for a set of annual monthly temperature data without consideration of seasonality in mean and variance. Therefore, it is not applicable to daily temperature data.

In daily temperature data, the quantile function should change periodically according to the season. To model such seasonality, as discussed in our exploratory analysis, we include the k-th order truncated Fourier series in the quantile function. Moreover, to investigate the impact of extreme climate events, the Southern Oscillation Index (SOI) is included as a covariate. The short term changes in Australia's climate are mostly associated with El Niño or La Niña events that indexed by the SOI. Including the SOI index in the model can remove the short-term natural variability from the long-term warming trend.

Next, we will describe our generalized model which is suitable for daily temperature data. It is worth noting that the model is specifically tailored for trend detection so that the time period t is confined within $[0,1]$ to satisfy the constraints of a quantile function $q(\tau|\mathbf{s},t)$ increasing in τ as suggested in Reich (2012). A model that is directly derived from Reich (2012) is as follows in Eq (8).

Let $0 = \kappa_1 < \ldots < \kappa_{L+1} = 1$ be a grid of equally spaced knots covering $[0,1]$. Then, for l with $\kappa_l < 0.5$,

$$B_l(\tau) = \begin{cases} \Phi^{-1}(\kappa_l) - \Phi^{-1}(\kappa_{l+1}) & \text{if} \quad \tau < \kappa_l \\ \Phi^{-1}(\tau) - \Phi^{-1}(\kappa_{l+1}) & \text{if} \quad \kappa_l \le \tau < \kappa_{l+1} \\ 0 & \text{if} \quad \kappa_{l+1} \le \tau \end{cases}$$

and, for l such that $\kappa_l \ge 0.5$,

$$B_l(\tau) = \begin{cases} 0 & \text{if} \quad \tau < \kappa_l \\ \Phi^{-1}(\tau) - \Phi^{-1}(\kappa_{l+1}) & \text{if} \quad \kappa_l \le \tau < \kappa_{l+1} \\ \Phi^{-1}(\kappa_{l+1}) - \Phi^{-1}(\kappa_l) & \text{if} \quad \kappa_{l+1} \le \tau \end{cases}.$$

Each quantile is assumed to be a function of time t for each station as follows:

$$q(\tau|\mathbf{s},t) = g_0(\tau|\mathbf{s}) + g_1(\tau|\mathbf{s})t + g_2(\tau|\mathbf{s})x_{soi} + FSk(t, \boldsymbol{g}_s(\tau|\mathbf{s}), \boldsymbol{g}_c(\tau|\mathbf{s})), \tag{8}$$

where $\boldsymbol{g}_s(\tau|\mathbf{s}) = (g_3(\tau|\mathbf{s}), \ldots, g_{k+2}(\tau|\mathbf{s}))$, $\boldsymbol{g}_c(\tau|\mathbf{s}) = (g_{k+3}(\tau|\mathbf{s}), \ldots, g_{2k+2}(\tau|\mathbf{s}))$, and for each k, $g_k(\tau|\mathbf{s})$ is taken to be linear combinations of L basis functions,

$$g_k(\tau|\mathbf{s}) = \beta_k(\mathbf{s}) + \sum_{l=1}^{L} B_l(\tau)\theta_{k,l}(\mathbf{s}). \tag{9}$$

The trend function $g_1(\tau|\mathbf{s})$ is of our particular interest for trend detection in this study. Note that when $g_k(\tau|\mathbf{s}) = 0$ for all $k > 1$, model in Eq (8) degenerates to a linear model that is exactly the same as that used in[19]. If further, we let $L = 1$ and $B_1(\mathbf{s}) = \Phi^{-1}(\tau)$, we obtain the special Gaussian case that corresponds to the model in[41].

Eq (8) can be further written as,

$$\begin{aligned} q(\tau|\mathbf{s},t) = &\underbrace{\beta_0(\mathbf{s}) + \beta_1(\mathbf{s})t + \beta_2(\mathbf{s})x_{soi} + FSk(t, \boldsymbol{\beta}_s(\mathbf{s}), \boldsymbol{\beta}_c(\mathbf{s}))}_{\mu_t(\mathbf{s})} \\ &+ \sum_{l=1}^{L} B_l(\tau) \underbrace{\left[\theta_{0,l}(\mathbf{s}) + \theta_{1,l}(\mathbf{s})t + \theta_{2,l}(\mathbf{s})x_{soi} + FSk(t, \boldsymbol{\theta}_{s,l}(\mathbf{s}), \boldsymbol{\theta}_{c,l}(\mathbf{s}))\right]}_{\sigma_l(\mathbf{s},t)}, \end{aligned} \tag{10}$$

where $\boldsymbol{\beta}_s(\mathbf{s}) = (\beta_i(\mathbf{s}), \ldots, \beta_{k+2}(\mathbf{s}))$, $\boldsymbol{\beta}_c(\mathbf{s}) = (\beta_{k+3}(\mathbf{s}), \ldots, \beta_{2k+2}(\mathbf{s}))$, $\boldsymbol{\theta}_{s,l}(\mathbf{s}) = (\theta_{3,l}, \ldots, \theta_{k+2,l})$, and $\boldsymbol{\theta}_{c,l}(\mathbf{s}) = (\theta_{k+3,l}, \ldots, \theta_{2k+2,l})$. In Eq (10), $\beta_k(\mathbf{s})$, the center of the quantile function at location $\mathbf{s}$, and $\theta_{k,l}(\mathbf{s})$ are unknown coefficients that determine the shape of the quantile function.

Here, adding these terms facilitates the model to account for seasonal heterogeneity in variance. However, auto-correlations in both mean and variance are not considered. Moreover, the variance function also depends on the mean values according to the exploratory analysis in Section . To account for all information, we further improve the model by replacing the $\theta_{0,l}(\mathbf{s}) + FSk(t, \boldsymbol{\theta}_{s,l}(\mathbf{s}), \boldsymbol{\theta}_{c,l}(\mathbf{s}))$ with $\theta_{2k+3,l}(\mathbf{s})\sigma_{d(t)}(\mathbf{s})$ in $\sigma_l(\mathbf{s},t)$, where $d(t)$ is the $d(t)$-th day of a year for the t-th time point and $\sigma_{d(t)}(\mathbf{s})$ is modeled in Eq (5). The new model is given as follows

$$\begin{aligned} q(\tau|\mathbf{s},t) = &\underbrace{\beta_0(\mathbf{s}) + \beta_1(\mathbf{s})t + \beta_2(\mathbf{s})x_{soi} + FSk(t, \boldsymbol{\beta}_s(\mathbf{s}), \boldsymbol{\beta}_c(\mathbf{s}))}_{\mu_t(\mathbf{s})} \\ &+ \sum_{l=1}^{L} B_l(\tau) \underbrace{\left[\theta_{1,l}(\mathbf{s})t + \theta_{2,l}(\mathbf{s})x_{soi} + \theta_{2k+3,l}(\mathbf{s})\sigma_{d(t)}(\mathbf{s})\right]}_{\sigma_l(\mathbf{s},t)}. \end{aligned} \tag{11}$$

Note that the model in Eq (11) is equivalent to adding a new term $g_{2k+3l}(\tau|\mathbf{s})\sigma_{d(t)}$ to Eq (10) and turning some unknown parameters ($\beta_{2k+3,l}(\mathbf{s})$, $\theta_{0,l}(\mathbf{s})$, $\boldsymbol{\theta}_{s,l}(\mathbf{s})$ and $\boldsymbol{\theta}_{c,l}(\mathbf{s})$) to zeros. Compared with the model in Eq (10), the model in Eq (11) has far fewer unknown parameters to estimate. In both spatio-temporal quantile models Eq (10) and (11), the quantile function can vary spatially by allowing both the $\beta_k(\mathbf{s})$ and $\theta_{k,l}(\mathbf{s})$ to be Gaussian spatial processes with exponential covariance. The β_k are independent Gaussian processes with mean $\bar{\beta}_k$ and covariance $COV(\beta_k(\mathbf{s}), \beta_k(\mathbf{s}')) = \psi^2_{\beta_k}\exp(-||\mathbf{s}-\mathbf{s}'||/\rho_{\beta_k})$. The $\theta_{k,l}$ are modeled similarly with mean $\bar{\theta}_{k,l}$ and covariance $COV(\theta_{k,l}(\mathbf{s}), \theta_{k,l}(\mathbf{s}')) = \psi^2_{\theta_k}\exp(-||\mathbf{s}-\mathbf{s}'||/\rho_{\theta_k})$, but they must satisfy $\sigma_l(\mathbf{s},t) > 0$ for all l and t.

The density function of $y_t(\mathbf{s})$ can be expressed in a closed form. Firstly, the quantile function can be written as

$$q(\tau|\mathbf{s},t) = \sum_{l=1}^{L}\left[a_l(\mathbf{s},t) + \sigma_l(\mathbf{s},t)\Phi^{-1}(\tau)\right] I_{\{\kappa_l \le \tau < \kappa_{l+1}\}}, \tag{12}$$

where $\sigma_l(\mathbf{s},t)$ is the coefficient of $B_l(\tau)$ and $a_l(\mathbf{s},t) = q(\kappa_{l+1}|\mathbf{s},t) - \sigma_l(\mathbf{s},t)\Phi^{-1}(\kappa_{l+1})$ if $\kappa_l < 0.5$ and $a_l(\mathbf{s},t) = q(\kappa_l|\mathbf{s},t) - \sigma_l(\mathbf{s},t)\Phi^{-1}(\kappa_l)$ if $\kappa_l \geq 0.5$. Then the density function of $y_t(\mathbf{s})$ is given as follows

$$f(y_t(\mathbf{s})) = \sum_{l=1}^{L} I_{\{q(\kappa_l|\mathbf{s},t) < y_t(\mathbf{s}) \leq q(\kappa_{l+1}|\mathbf{s},t)\}} N(y_t(\mathbf{s})|a_l(\mathbf{s},t), \sigma_l(\mathbf{s},t)^2), \tag{13}$$

where $\sigma_l(\mathbf{s},t) > 0$ for all l and t at each location $\mathbf{s}$.

Additional residual correlation is assumed to be an AR(1) process and handled with a copula approach using a latent residual process that is implemented in[19]. Let $v_t(\mathbf{s})$ be a latent Gaussian process modeled as follows,

$$\begin{aligned} v_1(\mathbf{s}) &= w_1(\mathbf{s}) \\ v_t(\mathbf{s}) &= \rho_v v_{t-1}(\mathbf{s}) + \sqrt{(1-\rho_v^2)} w_t(\mathbf{s}), \quad \text{for} \quad t > 1, \end{aligned} \tag{14}$$

where $|\rho_v| < 1$ and $w_t(\mathbf{s})$ are independent spatial process with mean 0 and covariance $COV(w_t(\mathbf{s}), w_t(\mathbf{s}')) = \exp(-||\mathbf{s}-\mathbf{s}'||/\psi_w)$. Here, $v_t(\mathbf{s}) \sim N(0,1)$ for each $\mathbf{s}$ and t, and let $u_t(\mathbf{s}) = \Phi(v_t(\mathbf{s})) \sim U(0,1)$. Let $\tau = u_t(\mathbf{s})$ in Eq (12); then we have

$$y_t(\mathbf{s}) = q(u_t(\mathbf{s})|\mathbf{s},t) = \sum_{l=1}^{L} \left[a_l(\mathbf{s},t) + \sigma_l(\mathbf{s},t) u_t(\mathbf{s})\right] I_{\{\kappa_l \leq u_t(\mathbf{s}) < \kappa_{l+1}\}}, \tag{15}$$

and

$$f(y_t(\mathbf{s})) = \sum_{l=1}^{L} I_{\{\kappa_l \leq u_t(\mathbf{s}) < \kappa_{l+1}\}} N(y_t(\mathbf{s})|a_l(\mathbf{s},t), \sigma_l(\mathbf{s},t)^2). \tag{16}$$

For model inference, parameters are estimated in a Bayesian manner via the Markov chain Monte Carlo method that was implemented in Reich (2012)[19]. Note that $\beta_k(\mathbf{s})$ and $\theta_{k,l}(\mathbf{s})$ are model parameters and randomly sampled from their prior distributions (which are Gaussian spatial process) using a Gibbs sampling approach. Here random samples of $\theta_{k,l}(\mathbf{s})$ must satisfy that $\frac{dq(\tau|\mathbf{s},t)}{d\tau} > 0$. Hype-parameters $\bar{\beta}_k(\mathbf{s})$ and $\bar{\theta}_{k,l}(\mathbf{s})$ are used to characterize the mean of prior distributions of $\beta_k(\mathbf{s})$ and $\theta_{k,l}(\mathbf{s})$, and ψ_{β_k}, ρ_{β_k}, ψ_{θ_k} and ρ_{θ_k} are used to characterize the variance. Uninformative priors for the hyper-parameters are used, where $\bar{\beta}_k$ and $\bar{\theta}_{k,l}$ have $N(0,10^2)$ priors, and ψ_{β_k}, ρ_{β_k}, ψ_{θ_k} and ρ_{θ_k} have $InvGamma(0.1,0.1)$ priors. In the residual correlation modeling, the auto-correlation ρ_v has a $U(-1,1)$ prior and ψ_w also has an $InvGamma(0.1,0.1)$ prior. Gibbs sampling is used for $\bar{\beta}_k(\mathbf{s})$, $\bar{\theta}_{k,l}(\mathbf{s})$ and ρ_v, while other spatial range hyper-parameters are updated by using Metropolis sampling with Gaussian candidate distribution (tuned to give roughly 40% acceptance rate).

Acknowledgements

The authors appreciated Professor Brian Reich for sharing the R code for the implementation of his spatio-temporal quantile regression model. The authors acknowledge the support of ARC Centre of Excellence for Mathematical & Statistical Frontiers (ACEMS) under the grant number CE140100049.

Data availability

Data is available on request from the corresponding author, and also can be obtained from the website of Bureau of Meteorology Australia and the `R` package `bomrang`.

Code availability

Code for data analysis is available on request from the corresponding author.

Author contributions

QD undertook the data collection and analysis, developed and implemented the methodology, and drafted the manuscript. CM developed the methodology and drafted the manuscript. GB initiated the study and reviewed the manuscript. KM reviewed and edited the manuscript. YGW designed the study, advised the methodology development, and reviewed and edited the manuscript.

Competing interests

The author(s) declare no competing interests.

References

1. Paik, S. & Min, S. K. Quantifying the anthropogenic greenhouse gas contribution to the observed spring snow cover decline using the cmip6 multi-model ensemble. *J. Clim.* (2020).
2. Spinoni, J. *et al.* Future global meteorological drought hot spots: A study based on cordex data. *J. Clim.* **33**, 3635–3661 (2020).
3. Stocker, T., Dahe, Q. & Plattner, G.-K. Climate change 2013: The physical science basis. intergovernmental panel on climate change (IPCC). (2013).
4. Pachauri, R. K. & Reisinger, A. (eds.) *Climate change 2007. Synthesis report. Contribution of Working Groups I, II and III to the fourth assessment report of the Intergovernmental Panel on Climate Change [Core Writing Team, Pachauri, R.K and Reisinger, A. (eds.)]* (IPCC, Geneva, Switzerland, 2007).
5. Pachauri, R. & Meyer, L. (eds.) *Climate Change 2014: Synthesis Report. Contribution of Working Groups I, II and III to the Fifth Assessment Report of the Intergovernmental Panel on Climate Change [Core Writing Team, R.K. Pachauri and L.A. Meyer (eds.)]* (IPCC, Geneva, Switzerland, 2014).
6. Sinha, B., Sévellec, F., Robson, J. & Nurser, A. J. G. Surging of Global Surface Temperature due to Decadal Legacy of Ocean Heat Uptake. *J. Clim.* **33**, 8025–8045 (2020).
7. Alexander, L. V. *et al.* Global observed changes in daily climate extremes of temperature and precipitation. *J. Geophys. Res. Atmospheres* **111** (2006).
8. Brown, S. J., Caesar, J. & Ferro, C. A. T. Global changes in extreme daily temperature since 1950. *J. Geophys. Res. Atmospheres* **113** (2008).
9. Zhang, P. *et al.* Observed changes in extreme temperature over the global land based on a newly developed station daily dataset. *J. Clim.* **32**, 8489–8509 (2019).
10. Tularam, A. & Mahbub, I. Time series analysis of rainfall and temperature interactions in coastal catchments. *J. Math. Stat.* **6**, DOI: 10.3844/jmssp.2010.372.380 (2010).
11. CSIRO & Bureau of Meteorology Australia. State of the climate 2020 (2020).
12. Bureau of Meteorology Australia. Annual climate statement 2019 (2019).
13. Ekström, M. *et al.* Central slopes cluster report, climate change in australia projections for australia's natural resource management regions: Cluster reports, eds. Tech. Rep., CSIRO and Bureau of Meteorology, Australia (2015).
14. Koenker, R. & Bassett, G. Regression quantiles. *Econometrica* **46**, 33–50 (1978).
15. Koenker, R. & Schorfheide, F. Quantile spline models for global temperature change. *Clim. Chang.* **28**, 395–404 (1994).
16. Barbosa, S. M., Scotto, M. G. & Alonso, A. M. Summarising changes in air temperature over central europe by quantile regression and clustering. *Nat. Hazards Earth Syst. Sci.* **11**, 3227–3233 (2011).
17. Gao, M. & Franzke, C. L. Quantile regression–based spatiotemporal analysis of extreme temperature change in china. *J. Clim.* **30**, 9897–9914 (2017).
18. Franzke, C. A novel method to test for significant trends in extreme values in serially dependent time series. *Geophys. Res. Lett.* **40**, 1391–1395 (2013).
19. Reich, B. J. Spatiotemporal quantile regression for detecting distributional changes in environmental processes. *J. Royal Stat. Soc. Ser. C (Applied Stat.* **61**, 535–553 (2012).
20. Bureau of Meteorology Australia. Climate information (australia) (2020).
21. Sparks, A. H., Padgham, M., Parsonage, H. & Pembleton, K. bomrang: Fetch australian government bureau of meteorology weather data. *The J. Open Source Softw.* **2** (2017).
22. Nicholls, Z. R. J. *et al.* Reduced complexity model intercomparison project phase 1: introduction and evaluation of global-mean temperature response. *Geosci. Model. Dev.* **13**, 5175–5190 (2020).
23. Wentworth Group of Concerned Scientists. *Assessment of river flows in the Murray-Darling Basin: Observed versus expected flows under the Basin Plan 2012-2019* (Sydney, Australia, 2020).

24. Yue, S., Pilon, P., Phinney, B. & Cavadias, G. The influence of autocorrelation on the ability to detect trend in hydrological series. *Hydrol. processes* **16**, 1807–1829 (2002).

25. Fatichi, S., Barbosa, S. M., Caporali, E. & Silva, M. E. Deterministic versus stochastic trends: Detection and challenges. *J. Geophys. Res. Atmospheres* **114** (2009).

26. Franzke, C. Long-Range Dependence and Climate Noise Characteristics of Antarctic Temperature Data. *J. Clim.* **23**, 6074–6081 (2010).

27. Franzke, C. Nonlinear Trends, Long-Range Dependence, and Climate Noise Properties of Surface Temperature. *J. Clim.* **25**, 4172–4183 (2012).

28. Maheu, J. Can garch models capture long-range dependence ? *Stud. Nonlinear Dyn. & Econom.* **9** (2005).

29. Moise, A. *et al.* Monsoonal north cluster report, climate change in australia projections for australia's natural resource management regions: Cluster reports, eds. Tech. Rep., CSIRO and Bureau of Meteorology, Australia (2015).

30. Timbal, B. *et al.* Murray basin cluster report, climate change in australia projections for australia's natural resource management regions: Cluster reports, eds. Tech. Rep., CSIRO and Bureau of Meteorology, Australia (2015).

31. Watterson, I. *et al.* Rangelands cluster report, climate change in australia projections for australia's natural resource management regions: Cluster reports, eds. Tech. Rep., CSIRO and Bureau of Meteorology, Australia (2015).

32. Grose, M. *et al.* Southern slopes cluster report, climate change in australia projections for australia's natural resource management regions: Cluster reports, eds. Tech. Rep., CSIRO and Bureau of Meteorology, Australia (2015).

33. Hope, P. *et al.* Southern and south-western flatlands cluster report, climate change in australia projections for australia's natural resource management regions: Cluster reports, eds. Tech. Rep., CSIRO and Bureau of Meteorology, Australia (2015).

34. McInnes, K. *et al.* Wet tropics cluster report, climate change in australia projections for australia's natural resource management regions: Cluster reports, eds. Tech. Rep., CSIRO and Bureau of Meteorology, Australia (2015).

35. CSIRO & Bureau of Meteorology Australia. State of the climate 2018 (2018).

36. Campbell, S. D. & Diebold, F. X. Weather forecasting for weather derivatives. *J. Am. Stat. Assoc.* **100**, 6–16 (2005).

37. Benth, F. E. & Benth, J. Š. The volatility of temperature and pricing of weather derivatives. *Quant. Finance* **7**, 553–561 (2007).

38. Benth, J. Š., Benth, F. E. & Jalinskas, P. A spatial-temporal model for temperature with seasonal variance. *J. Appl. Stat.* **34**, 823–841 (2007).

39. Sirangelo, B., Caloiero, T., Coscarelli, R. & Ferrari, E. A stochastic model for the analysis of maximum daily temperature. *Theor. Appl. Climatol.* **130**, 275–289 (2017).

40. Benth, F. E. & Benth, J. Š. Stochastic modelling of temperature variations with a view towards weather derivatives. *Appl. Math. Finance* **12**, 53–85 (2005).

41. Chandler, R. E. On the use of generalized linear models for interpreting climate variability. *Environmetrics* **16**, 699–715 (2005).

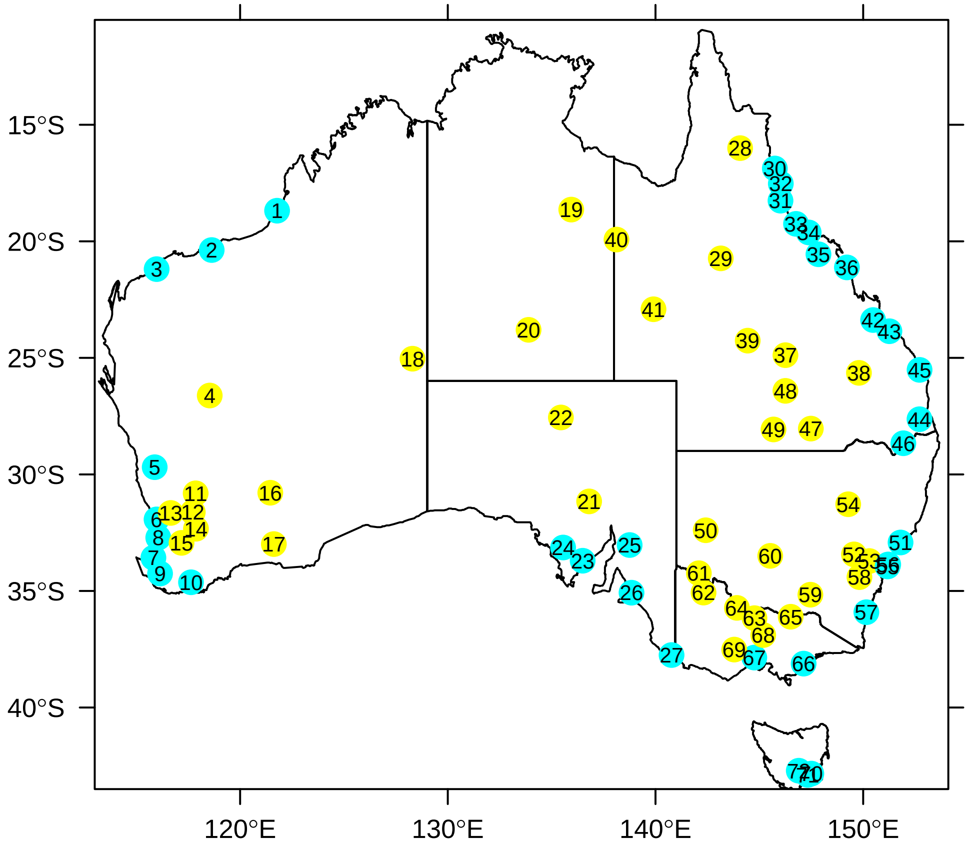

Figure 1. The distribution of selected stations with qualifying data. Blue dots are for (near) coastal stations and yellow dots are for inland stations.

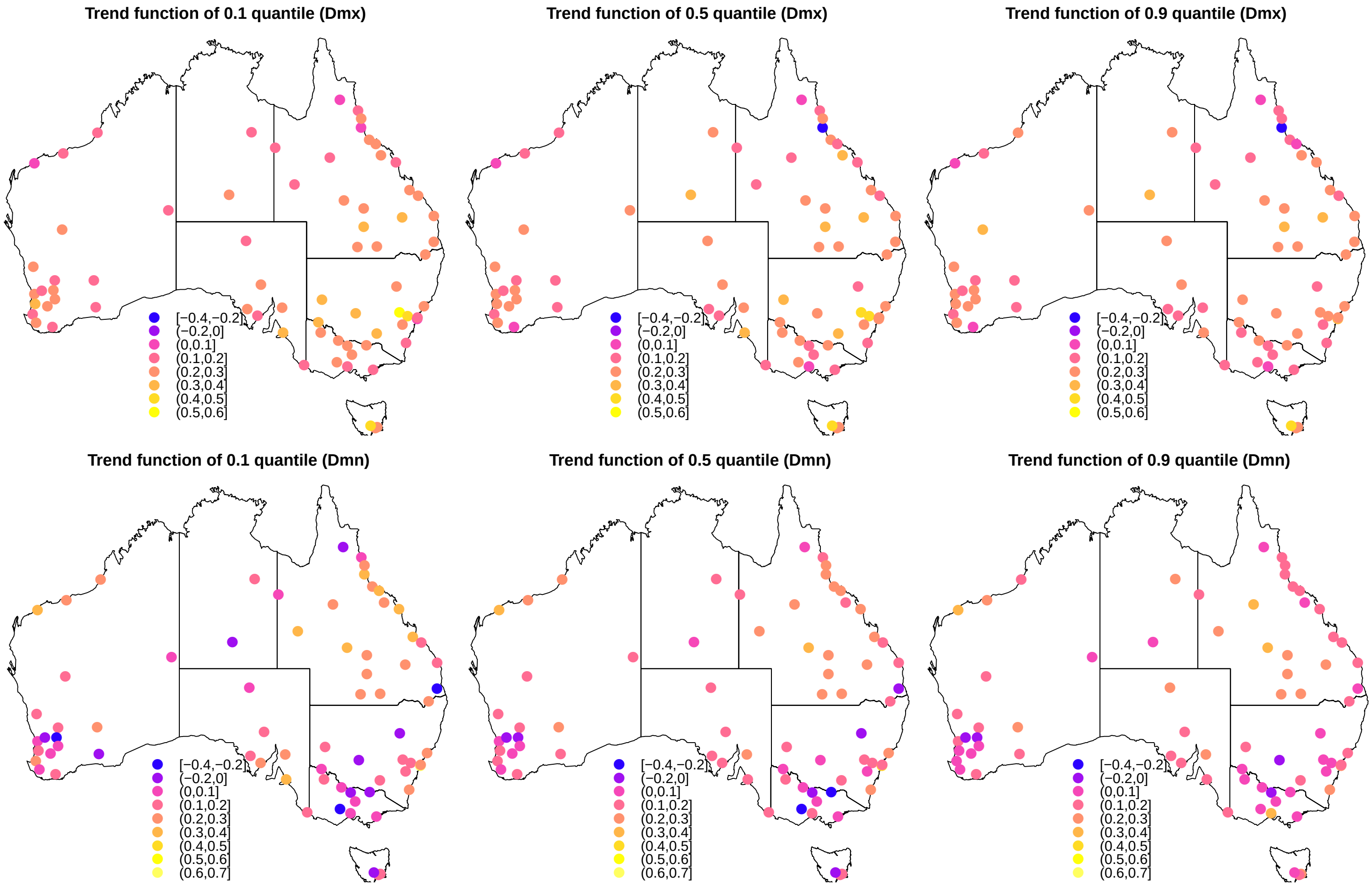

Figure 2. Quantile trend of $\tau = 0.1, 0.5$ and 0.9 for all 72 stations during the study period. The color of point is the total degrees Celsius that increased/decreased per decade from 1960 to 2019. The top panels are for the Dmx series and the bottom panels are for the Dmn series.

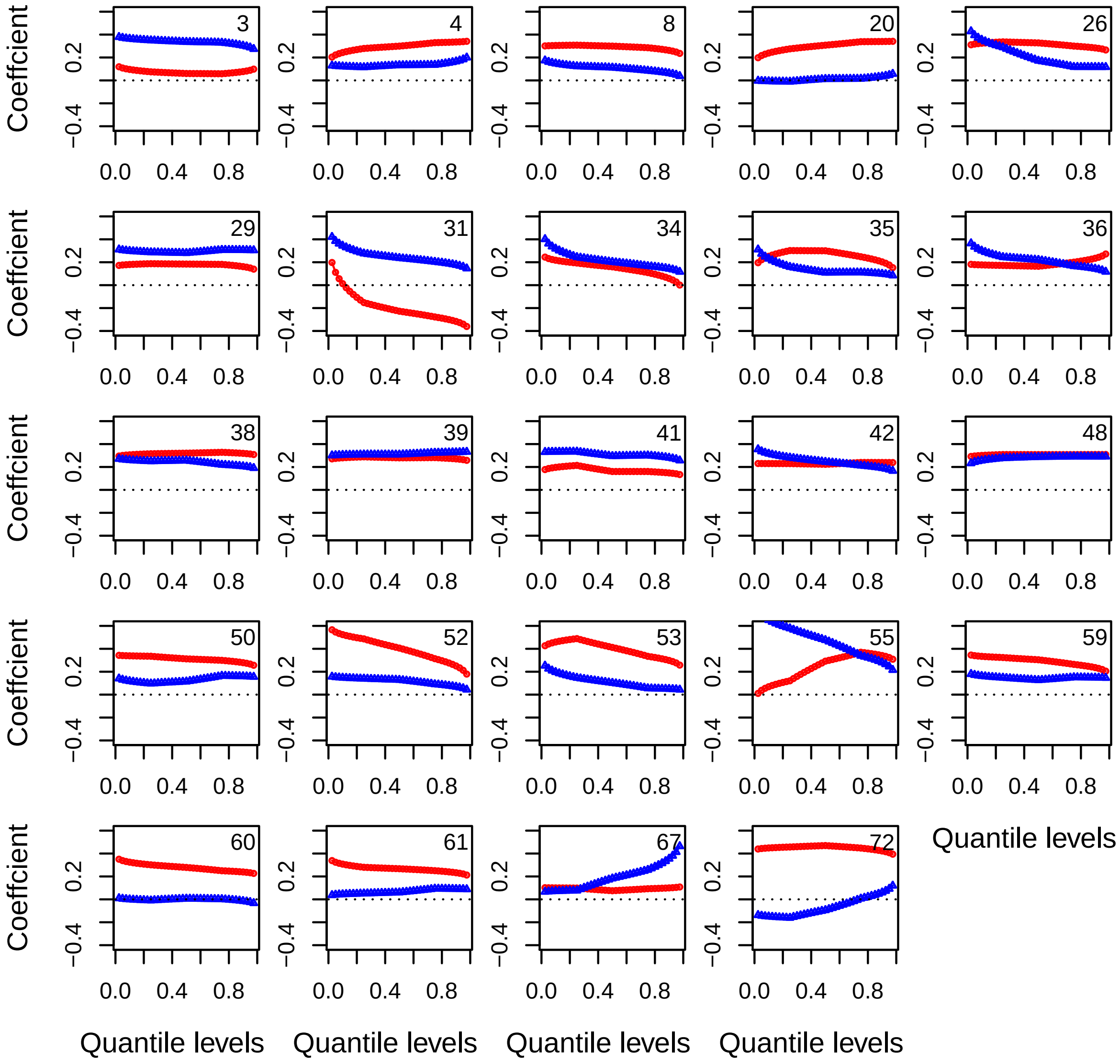

Figure 3. Quantile trend as a function of quantile level for selected stations. The red curve is for Dmx and the blue curve for Dmn.

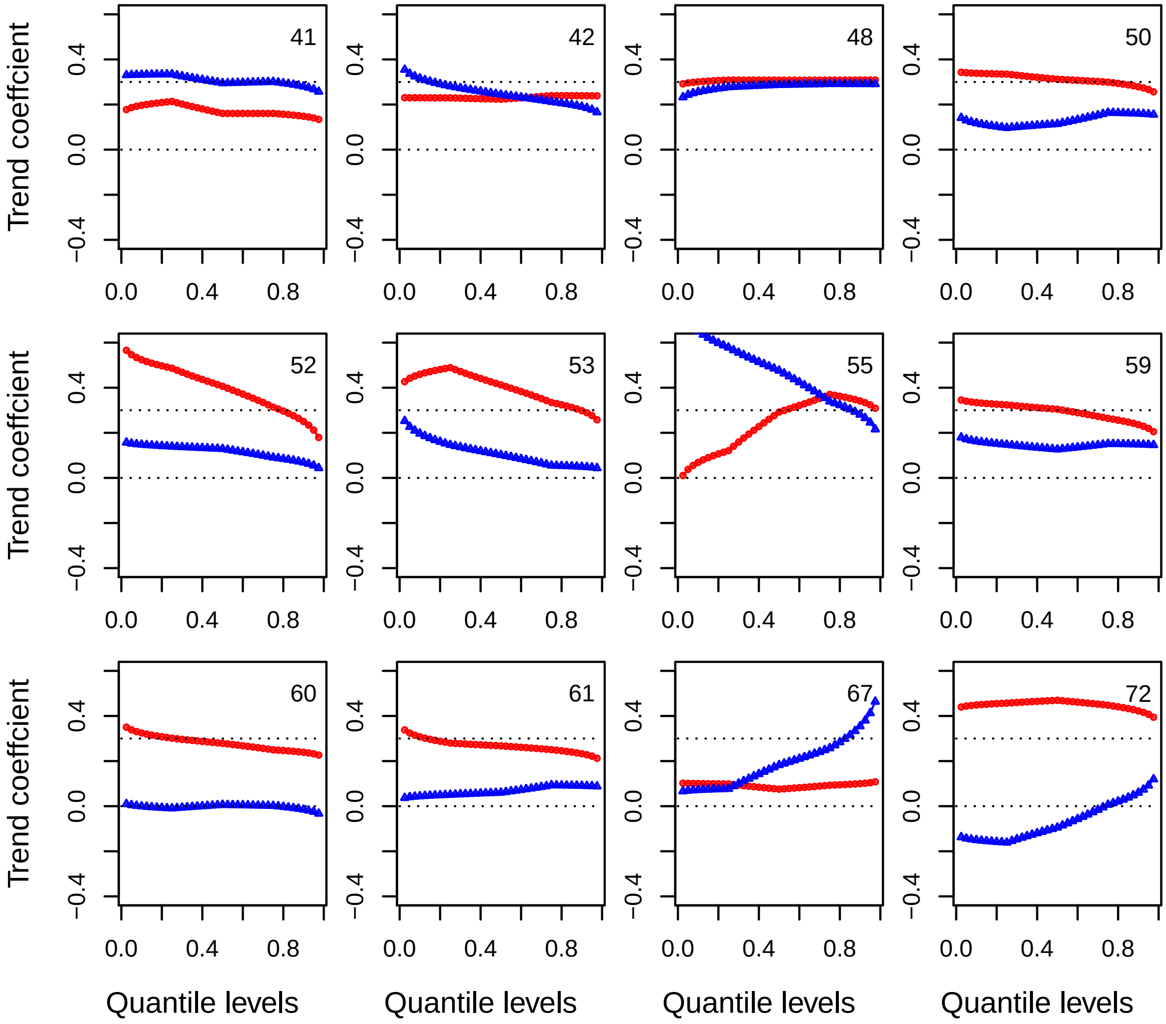

Figure 4. Quantile trend as a function of quantile level for selected stations. The red curve is for Dmx and the blue curve for Dmn.

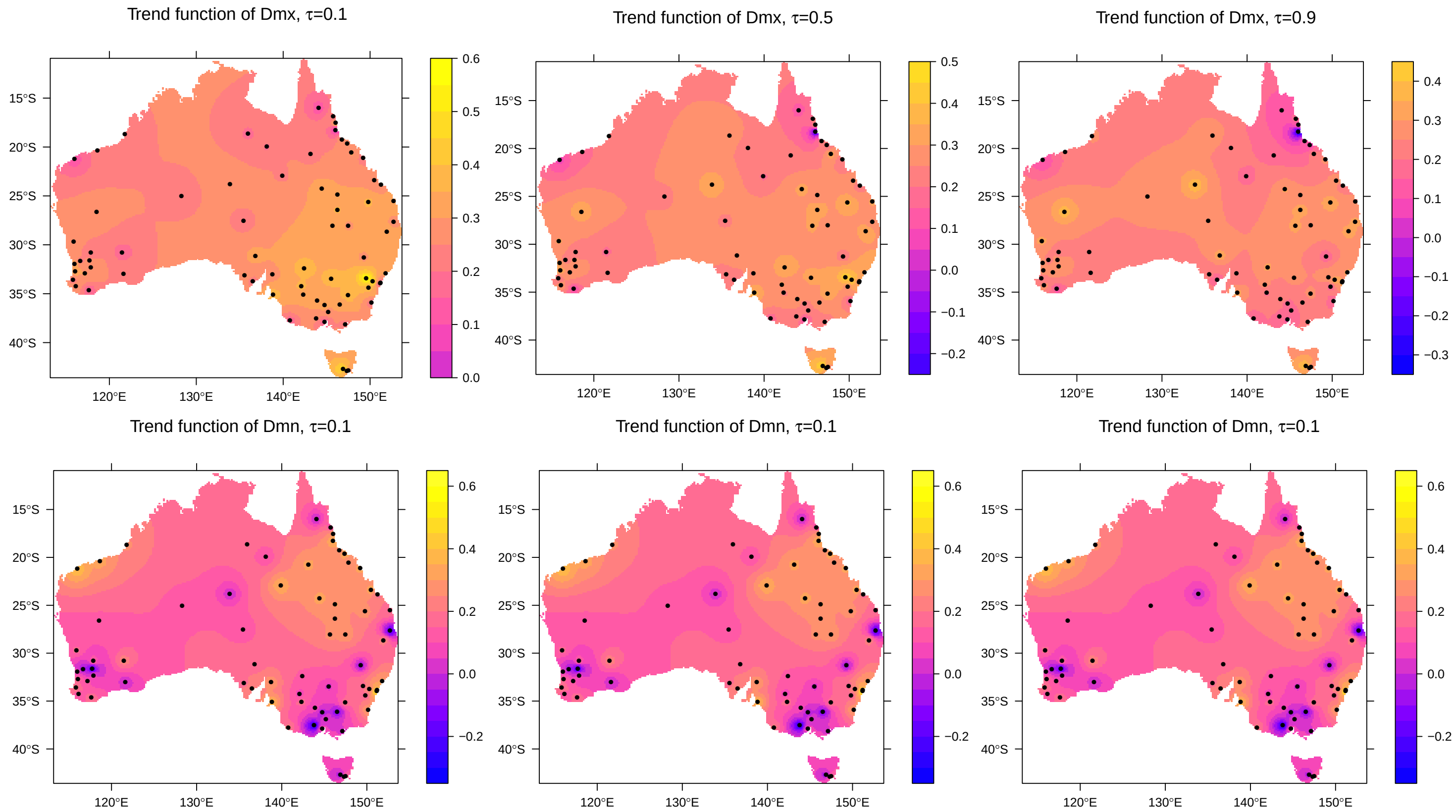

Figure 5. Quantile trend of $\tau = 0.1, 0.5$ and 0.9 for overall Australia during the study period. The color of point is the total degrees Celsius that increased/decreased per decade from 1960 to 2019. The top panels are for the Dmx series and the bottom panels are for the Dmn series.

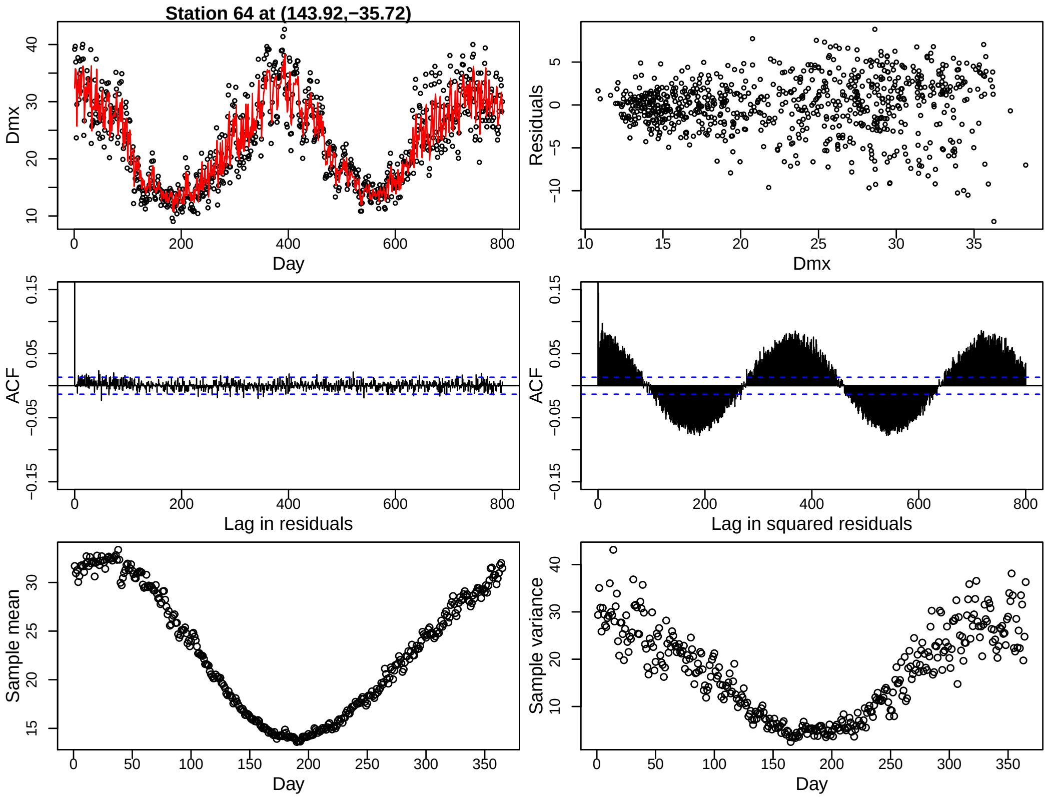

Figure 6. Exploratory analysis for a single station. Top left: fitting parametric mean model for Dmx. Top right: residuals of fit against predicted Dmx for the parametric mean model. Middle left: Auto-Correlation Function (ACF) plot for residuals for lag up to 800 days. Middle right: ACF plot for squared residuals for lag up to 800 days. Bottom left: sample mean over 60 years. Bottom right: sample variance over 60 years.
